# Is it RSV?

**DOI:** 10.1002/hsr2.97

**Published:** 2018-10-12

**Authors:** Christopher S. Ambrose

**Affiliations:** ^1^ AstraZeneca Pharmaceuticals One MedImmune Way Gaithersburg Maryland USA

## Abstract

A study of infant bronchiolitis–coded episodes described the proportion of events attributable to respiratory syncytial virus (RSV) and demonstrated that episodes occurring during the peak months of winter viral season, among younger infants, and among those with higher levels of care, were more likely to be attributable to RSV.

## Abstract 2

This article discusses: Prevalence of infant bronchiolitis‐coded healthcare encounters attributable to RSV

In their article, “Prevalence of Infant Bronchiolitis–coded Healthcare Encounters Attributable to RSV,” Turi et al used data from Kaiser Permanente Northern California (KPNC) databases to describe the proportion of infant bronchiolitis–coded episodes attributable to RSV, as a function of various clinical and environmental factors.^1^ Their work represents a valuable contribution to the existing medical literature regarding the burden of RSV in infants and will help inform future RSV research that relies upon health‐care utilization data classified by International Classification of Disease (ICD) codes.

Researchers using ICD‐coded data to describe health‐care utilization due to respiratory viral disease face a difficult challenge: ICD codes cannot tell them precisely which events are due to a specific virus. Although virus‐specific codes are available for the most common viral pathogens, and those codes are utilized by providers, patients with those codes may or may not have had laboratory testing to confirm the presence of the specific virus. Additionally, providers can use “unspecified” codes even if laboratory testing was conducted and a pathogen was identified. As a result, researchers must interpret virus‐specific codes that may not be accurate and that underrepresent the true incidence of disease along with unspecified codes that have decreased and unknown specificity for the disease. This problem is particularly acute within RSV research given current recommendations against routine laboratory testing for RSV.^2^

In their analysis of ICD‐coded events and RSV testing in KPNC infants, Turi et al demonstrated that of all bronchiolitis episodes that were tested, 54% were RSV positive. Consistent with the statements above, of the RSV‐coded bronchiolitis and pneumonia episodes with an RSV test, 93% and 73% were positive for RSV, respectively, whereas 46% of unspecified bronchiolitis episodes with an RSV test were RSV positive. Factors linked to higher RSV positivity included those known to be associated with an increased risk of disease caused by RSV relative to other causes of bronchiolitis, specifically young infant age, occurrence during the RSV season, and greater level of care (hospital > emergency department [ED] > outpatient). Importantly, as the authors noted, their observations must be interpreted with the knowledge that RSV testing was not done systematically; providers were more likely to conduct RSV testing with younger infants, with preterm infants, in the hospital setting, and during the RSV season.

To best determine the proportions of ICD‐coded events that are due to RSV versus other etiologies, a study ideally would systematically test infants presenting with respiratory illness without influencing provider treatment or coding. Two recent studies referenced by the authors, Makari et al^3^ and Hall et al,^4^ meet these criteria. However, these studies had limitations: the study by Makari et al was limited to ED encounters in specific peak and shoulder months of the RSV season, and the study by Hall et al was limited to four US centers and did not examine ICD‐coded events in detail.

Despite their limitations, the studies by Turi et al, Makari et al, and Hall et al can collectively provide evidence‐based estimates for the proportions of ICD‐coded events that are truly due to RSV. Among infants, these studies found that approximately 90% of RSV‐coded events were RSV positive upon testing,^1^, ^3^ supporting the high specificity of RSV‐specific ICD codes. Additionally, depending on month and level of care, approximately 30% to 60% of events coded as unspecified bronchiolitis were RSV positive upon testing.^1^, ^3^ In Makari et al, among infants with an ED visit confirmed to be due to RSV with testing, 25% to 35% had an RSV‐specific ICD code and 65% to 79% had a bronchiolitis ICD code.^3^ Similarly, in Hall et al, among infants with laboratory‐confirmed RSV hospitalization, 53% had a discharge diagnosis of RSV and 85% had a discharge diagnosis of bronchiolitis.^4^ On the basis of these estimates, a picture of RSV prevalence among ICD‐coded events can be assembled (Figure 1), with the important knowledge that the precise positions and sizes of the categories will vary on the basis of child age, calendar month, and level of care. Additional variability will occur on the basis of geographic, seasonal, and temporal differences in provider coding, RSV testing, and the circulation of RSV relative to other respiratory viruses. Because of these latter sources of variability, precise generalizable estimates for the relationships depicted in Figure 1 are not possible. However, the observations of Turi et al, Makari et al, and Hall et al have given us consistent approximations that improve our understanding of US health‐care utilization associated with RSV disease and can inform future research and public health policy.

**Figure 1:**
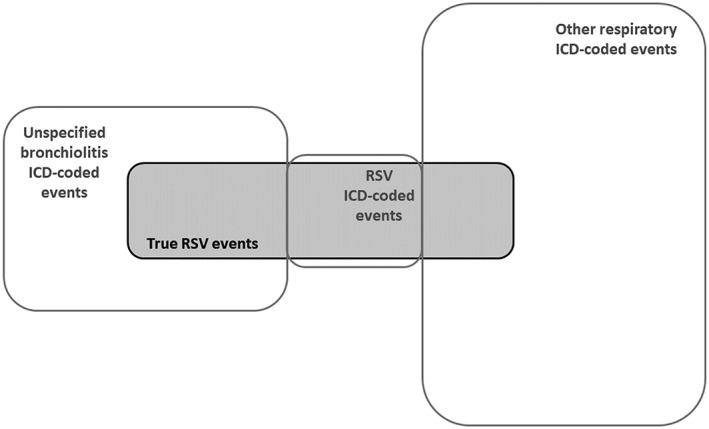
General relationships between true respiratory syncytial virus (RSV) disease and International Classification of Disease (ICD)–coded events among US infants, based on three recent studies

## CONFLICTS OF INTEREST

Christopher S. Ambrose is an employee of AstraZeneca, the manufacturer of palivizumab.
